# Peptide binding to HLA-DP proteins at pH 5.0 and pH 7.0: a quantitative molecular docking study

**DOI:** 10.1186/1472-6807-12-20

**Published:** 2012-08-05

**Authors:** Atanas Patronov, Ivan Dimitrov, Darren R Flower, Irini Doytchinova

**Affiliations:** 1 School of Pharmacy, Medical University of Sofia, 2 Dunav st, Sofia, 1000, Bulgaria; 2 Life and Health Sciences, Aston University, Aston Triangle, Birmingham, B4 7ET, UK

## Abstract

**Background:**

HLA-DPs are class II MHC proteins mediating immune responses to many diseases. Peptides bind MHC class II proteins in the acidic environment within endosomes. Acidic pH markedly elevates association rate constants but dissociation rates are almost unchanged in the pH range 5.0 – 7.0. This pH-driven effect can be explained by the protonation/deprotonation states of Histidine, whose imidazole has a pK_a_ of 6.0. At pH 5.0, imidazole ring is protonated, making Histidine positively charged and very hydrophilic, while at pH 7.0 imidazole is unprotonated, making Histidine less hydrophilic. We develop here a method to predict peptide binding to the four most frequent HLA-DP proteins: DP1, DP41, DP42 and DP5, using a molecular docking protocol. Dockings to virtual combinatorial peptide libraries were performed at pH 5.0 and pH 7.0.

**Results:**

The X-ray structure of the peptide – HLA-DP2 protein complex was used as a starting template to model by homology the structure of the four DP proteins. The resulting models were used to produce virtual combinatorial peptide libraries constructed using the single amino acid substitution (SAAS) principle. Peptides were docked into the DP binding site using AutoDock at pH 5.0 and pH 7.0. The resulting scores were normalized and used to generate Docking Score-based Quantitative Matrices (DS-QMs). The predictive ability of these QMs was tested using an external test set of 484 known DP binders. They were also compared to existing servers for DP binding prediction. The models derived at pH 5.0 predict better than those derived at pH 7.0 and showed significantly improved predictions for three of the four DP proteins, when compared to the existing servers. They are able to recognize 50% of the known binders in the top 5% of predicted peptides.

**Conclusions:**

The higher predictive ability of DS-QMs derived at pH 5.0 may be rationalised by the additional hydrogen bond formed between the backbone carbonyl oxygen belonging to the peptide position before p1 (p-1) and the protonated ε-nitrogen of His^79β^. Additionally, protonated His residues are well accepted at most of the peptide binding core positions which is in a good agreement with the overall negatively charged peptide binding site of most MHC proteins.

## Background

Major histocompatibility complex class II (MHC class II) proteins are normally found in B lymphocytes, dendritic cells, and macrophages; they are primarily involved in processing foreign, extracellular antigens, which are endocytozed and then enclosed in endosomes containing acid proteases. The pH in endosomes ranges from 4.5 to 6.0 [1]. In endosomes, antigens are degraded into oligopeptides. MHC class II proteins are synthesized in the endoplasmic reticulum (ER) and bind to a protein known as the MHC class II-associated invariant chain (Ii). Ii facilitates the export from the ER of MHC class II proteins and prevents binding to peptides resident in the ER. The complex MHC-Ii enters a specific endocytic compartment, called MIIC (MHC class II compartment), which fuses with endosomes. Ii is cleaved initially to the so-called CLIP fragment, with CLIP later being displaced by high-affinity peptides. The peptide – MHC class II protein complex is translocated to the cell surface, where it is recognized by CD4+ T cells.

Peptides binding to MHC class II vary in length from 12 to 25 amino acids, yet the binding site accepts only nine peptide residues, the rest extending from both ends as the cleft is open-ended. The side chains of bound peptides project into several binding pockets while a system of hydrogen bonds forms between the peptide backbone and the side chain atoms of the MHC [2].

Human MHCs, known as HLA (Human Leukocyte Antigens), are extremely polymorphic and polygenic. The IMGT/HLA database lists over 1,600 HLA class II proteins [3]. HLAs class II contain three loci: DR, DQ and DP. DR and DQ proteins are well studied, while DP was initially considered of lesser importance in immune responses. However, it is now clear that HLA-DP proteins have important roles in mediating the immune response to many diseases, such as graft-versus-host (GVH) disease [4], sarcoidosis [5], juvenile chronic arthritis [6], Graves’ disease [7], hard metal lung disease [8] and especially, chronic beryllium disease [9]. Recently, the X-ray structure of the HLA-DP2 (DPA*0103, DPB1*0201) in complex with a self-peptide derived from the HLA-DR α-chain has been determined [10]. Although the overall structure of DP2 is similar to that of other MHC class II proteins, it contains a unique solvent-exposed acidic pocket containing three glutamic acids (Glu^26β^, Glu^68β^ and Glu^69β^). This pocket may be able to bind Beryllium and present it to T cells, providing a mechanistic explanation that underlies chronic Beryllium disease [10,11]. X-ray data also reveals that the DP2 binding site comprises four binding pockets: deep, hydrophobic pockets p1 and p6; large, shallow, negatively charged p4; and deep, narrow and polar pocket p9.

Peptides bind to MHC class II proteins in an acidic environment (pH ~ 5.0). Bell-shaped profiles with optima at pH 5.0 are observed in many peptide – MHC class II binding experiments [12-14]. Acidic pH markedly elevated association rate constants 40 fold; dissociation rates are, by contrast, almost unchanged in the pH range 5.0 – 7.0 [13]. The equilibrium binding level is thus enhanced at pH 5.0. The influence of pH on the binding equilibrium can be explained by subtle conformational changes due to altered protonation and deprotonation states and near neighbor interactions. The only amino acid sensitive to pH in the range 5.0 – 7.0 is histidine. The side-chain pK_a_ of the His imidazole is 6.0. At pH 5.0 imidazole is protonated and His is thus positively charged and very hydrophilic. At pH 7.0, imidazole is unprotonated making His less hydrophilic. Thus, a pair of amino acids consisting of His and a hydrophobic residue could function as a pH-sensitive “His button” [14]. It “closes” at pH 7.0 (hydrophobic interaction) and “opens” at pH 5.0 (hydrophobic – charge repulsion). Such pH-sensitive switch was observed for His^33α^ in the formation of HLA-DR1 – HLA-DM complexes [14].

There are five His residues in the HLA-DP binding cleft: four belong to the α-chain (positions 5, 16, 44 and 79) and one to the β-chain (position 79). All five histidine residues are conserved among DP proteins. His^79β^ side chain contacts the binding peptide in the vicinity of peptide position 2. Recently, a favorable π-π stacking between the aromatic rings of His^79β^ and His^2peptide^ was identified [15]. The other His residues are remote from the binding site and do not make contact with the bound peptide.

Molecular docking is a key structure-based method with significant utility in drug design, bioinformatics, and immunoinformatics. In contrast to sequence-based approaches, virtual docking experiments do not require extensive pre-existing experimental data. The only information necessary is a reliable model of the peptide – MHC protein complex, as provided by X-ray crystallography. Docking methodology allows the development of predictive models where the training and test data are fully independent, thus, eliminating any possibility of over-fitting. We use rigid docking to identify optimised bound peptide conformations; since even for a nonamer, a fully unconstrained peptide docking would be of a prohibitively extended duration. However, since the number of distinct peptide conformations observed within currently-known X-ray structures remains very small, we make the parsimonious and wholly-reasonable assumption that peptides will bind in a similar conformation. Molecular docking has been extensively and rigorously tested on both peptide-MHC class I and peptide-MHC class II complexes. As an approach to evaluating peptide binding to MHCs, it has proved to be rapid, accurate, and reliable [15-17].

Recently, we applied a molecular docking protocol to a library of modeled peptide-DP2 complexes to assess the contribution of each of the 20 naturally occurred amino acids at each of the nine binding core positions and four flanking residues (two at both ends) [15]. The normalized binding scores formed a quantitative matrix (QM), known also as a position-specific scoring matrix (PSSM). PSSMs are a commonly used representation of motifs or patterns within biological sequences. The predictive ability of the derived QM was assessed using an external test set of known binders to DP2. A comparison to predictions made by existing servers for DP2 binding prediction indicated an improvement in performance offered by our docking score-based QM (DS-QM) [15].

In the present study, we modelled by homology four of the most frequent HLA-DP proteins [18]: DP1 (DPA1*0201/DPB1*0101), DP41 (DPA1*0103/ DPB1*0401), DP42 (DPA1*0103/DPB1*0402) and DP5 (DPA1*0201/DPB1*0501). We applied a similar docking protocol to derive DS-QMs for peptide binding prediction [15]. To investigate the influence of pH on the predictive ability, different QMs were derived at two pH values: 5.0 and 7.0. The QMs were validated using external test sets and compared to other servers for DP binding prediction. Additionally, in order to analyze the peptide-MHC protein interaction interface, a single docking of HLA-DP2 (DPA*0103, DPB1*0201) in complex with a self-peptide derived from the HLA-DR α-chain (pdb code: 3lqz) was analyzed using Rosetta Dock [19]. Our analysis affords a deep and detailed analysis of the different amino acid preferences at each position of peptides binding DP proteins.

## Methods

### Input data

The X-ray structure of the HLA-DP2 (DPA*0103, DPB1*0201) protein, in complex with a self-peptide derived from the HLA-DR α-chain, was used as the starting structure for homology modelling [10]. The covalently bound peptide was separated and defined as chain C. It consists of nine binding core positions (FHYLPFLPS) and six flanking residues (RK at the N terminus and TGGS at the C terminus). The conformation of the protein was used to model by homology the four HLA-DP proteins. The conformation of the bound peptide was used as a template for the modelling of four virtual combinatorial peptide libraries.

### Homology modelling

Models of four HLA-DP proteins were built using the X-ray structure of HLA-DP2 protein (pdb code: 3lqz) as the template for homology modelling. HLA-DP proteins used were: DP1 (DPA1*0201/DPB1*0101), DP41 (DPA1*0103/ DPB1*0401), DP42 (DPA1*0103/DPB1*0402), and DP5 (DPA1*0201/DPB1*0501). The polymorphic amino acids among the first 80 amino acids from chain α (DPA1) and the first 90 amino acids from chain β (DPB1) were mutated accordingly. The resulting structure, in complex with the native peptide from the starting X-ray structure, was subjected to energy minimization by simulated annealing using the AMBER force field [20]. Each peptide-DP protein complex was used as a starting structure for generating the corresponding virtual peptide library.

### Combinatorial peptide library

The nine positions forming the peptide binding core were examined. Four peptide libraries, each consisted of 172 peptides (19 amino acids × 9 positions + 1 original ligand), were built using PyMOL [21]. The SAAS (single amino acid substitution) approach was used to model the conformations of each altered side chains: after substitution, the peptide was minimized while keeping the MHC protein rigid. The protonation state of ionisable protein side chains was assigned to a standard ionisable state: neutral for His; positively charged for Arg and Lys; and negatively charged for Asp and Glu [22]. In the case of docking at pH 5, His was considered to be positively charged.

### AutoDock protocol

A parallelized version of AutoDock 4.2 [23], employing an implementation of the Lamarckian genetic algorithm (GA), was used to model the peptide binding to HLA-DPs. All simulations were run on the IBM Blue Gene – P of the Bulgarian Supercomputing Centre. The input ligands for AutoDock 4.2 were prepared by using tools developed in-house using C# and .NET. The output data were mined by python scripts using the MGL Tools 1.5.4 package [24]. All retained poses considered in the study had an RMSD below 2.0 Å. To limit the computational burden of calculating peptide–MHC interactions at positions not involved in the static docking, all coordinates were kept fixed apart from the peptide residues of interest. These were left flexible. All GA settings were kept to their default values, apart from the number of energy evaluations and the number of generations which were set to 2 500 000 and 27 000, respectively. The docking grid was defined as a cuboid with respective dimensions of: 68 Å × 80 Å × 80 Å for DP1, 72 Å × 80 Å × 82 Å for DP41, 72 Å × 80 Å × 82 Å for DP42 and 72 Å × 80 Å × 82 Å for DP5 which encompassed the entire peptide binding site on DP. The output from ten independent GA runs for each ligand was processed and the pose (binding conformation) with the lowest Free Energy of Binding (FEB) was considered. FEB values represent the direct output from the AutoDock 4.2 scoring function which takes into consideration weighted terms for van der Waals dispersion/repulsion, hydrogen bonding, electrostatics, desolvation interactions, and the change in torsional free energy when the ligand goes from an unbound to a bound state.

### Docking score-based quantitative matrices (DS-QMs)

The FEBs derived from the docking experiments had negative and positive values. Negative FEBs correspond to binding peptides, while positive FEBs correspond to non-binding peptides. Only negative FEBs were considered; non-binding amino acids were assigned the penalty score of −10. The FEBs were normalized position per position using the following formula:

(1) $$FEB_{i,norm}=\frac{FEB_{i}-\bar{FEB}}{FEB_{\max}-FEB_{\min}}\text{,}$$

Where FEB_i_ is the binding energy of the i-th peptide, $\bar{FEB}$ is the average for a given position, FEB_max_ and FEB_min_ are the maximum and minimum FEBs for a given position. Normalized FEBs were multiplied by (−1) before being entered into the quantitative matrices (QMs) for ease of presentation. Thus, the positive FEBs correspond to preferred amino acids, and negative FEBs to non-preferred residues. Eight QMs were derived: two for each HLA-DP protein at pH 5.0 and pH 7.0, respectively.

### Test set

Four test sets of peptides known to bind HLA-DP1, HLA-DP41, HLA-DP42, and HLA-DP5, respectively, were collected from the Immune Epitope Database [25] (June 2011 release). The test set of DP1 binders contained 102 peptides originating from 60 proteins. The DP41 test set contained 152 binding peptides from 71 proteins. The DP42 test set contained 122 binding peptides from 66 proteins. The DP5 test set contained 108 binding peptides from 66 proteins. The peptides had different lengths. No multiple binders were used. Each protein was represented as a set of overlapping nonamers. The nonameric subsequence of any known binder with the highest score was considered a binder; all other protein nonamers were considered as non-binders. The binding score of each nonamer was calculated as a sum of the weights of all nine positions or of different combinations thereof.

The tests were performed under conditions similar to those which an experimental immunologist might use: proteins were cleaved into overlapping nonamers, the binding score of each nonamer was predicted. Nonamers were then ranked according to their binding score and the top 5% of the predicted nonamers was selected. The selected peptides were then compared to the known binders. If the nonamer sequence was part of the known binder sequence, the predicted peptide was considered as a true predicted binder. The ratio of all true predicted binders to all binders in the corresponding test set defined the *sensitivity* of prediction at the top 5% cut-off. The test sets used in the present study are given as Additional file 1.

Additionally, the models were compared in terms of the *area under the receiver operating characteristics curve (AUC)*. Two variables - *sensitivity* and *1-specificity* - were calculated at different thresholds. *AUC* is a quantitative measure of predictive ability and varies from 0.5 for random prediction to 1.0 for a perfect prediction.

### Rosetta Dock protocol

The Rosetta Dock server ( http://rosettadock.graylab.jhu.edu) was used to generate the pair interaction energies across the peptide-DP2 protein binding interface. The X-ray structure of the peptide-HLA-DP2 (DPA*0103, DPB1*0201) complex (pdb code: 3lqz) was used as input. The RosettaDock output file contains a table of pair energies across the binding interface. Several energy terms are generated: *E*_*tot*_ is the sum of all energies between the pair residues; *E*_*atr*_ and *E*_*rep*_ are the Lennard – Jones attractive and repulsive energies, respectively; *E*_*sol*_ is the solvation energy according to the Lazaridis–Karplus solvation model [26], which penalizes buried polar groups; *E*_*hbnd*_ is the hydrogen bonding energy per residue; *E*_*pair*_ is a statistically-based pair term derived from the PDB database, which favours salt bridges.

## Results

### Pair energies across peptide – HLA-DP2 protein binding interface

The peptide-DP protein binding interface was analysed using the RosettaDock server [19].

**Figure 1:**
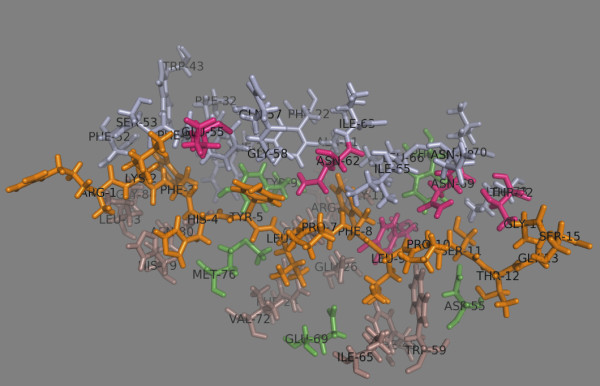
**Peptide binding site on HLA-DP2.** Residues from the α chain are shown in light blue. Residues from the β chain are shown in darksalmon. The polymorphic residues are shown in green. Residues making H-bonds with the peptide amino acids are shown in magenta. The bound peptide is shown in orange.

It consists of 39 residues: 21 residues belong to α-chain (9, 11, 22, 24, 32, 43, 52, 53, 54, 55, 57, 58, 62, 63, 65, 66, 68, 69, 70, 72 and 73) and 18 residues are from β-chain (9, 11, 12, 13, 24, 26, 28, 45, 55, 59, 65, 69, 72, 76, 79, 80, 83 and 84) [10] (Figure 1). Only five of the residues are polymorphic among the five most frequent DP proteins (Table 1). These are Tyr/Phe^9β^, Ala/Asp/Glu^55β^, Lys/Glu^69β^, Val/Met^76β^ and Asp/Gly^84β^. Asp^55β^ is involved in a salt-bridge with peptide Ser9, while the other polymorphic residues do not form either an H-bond or a salt bridge with the bound peptide.

**Table 1:** Alignment of HLA-DPA1 (α chain) and HLA-DPB1 (β chain) for the five most frequent DP proteins

AA Pos.	10	20	30	40	50	60
DPA1*01:03	IKADHVSTYA	AFVQTHRPTG	EFMFEFDEDE	MFYVDLDKKE	TVWHLEEFGQ	AFSFEAQGGL
DPA1*02:01	----------	----------	----------	Q---------	---------R	----------
AA Pos.	70	80				
DPA1*01:03	ANIAILNNNL	NTLIQRSNHT				
DPA1*02:01	----------	----------				
AA Pos.	10	20	30	40	50	60
DPB1*01:01	RATPENYVYQ	GRQECYAFNG	TQRFLERYIY	NREEYARFDS	DVGEFRAVTE	LGRPAAEYWN
DPB1*02:01	-------LF-	----------	----------	----LF----	----------	----DE----
DPB1*04:01	-------LF-	----------	----------	-----F----	----------	----------
DPB1*04:02	-------LF-	----------	----------	----FV----	----------	----DE----
DPB1*05:01	-------LF-	----------	----------	----LV----	----------	----E-----
AA Pos.	70	80	90			
DPB1*01:01	SQKDILEEKR	AVPDRVCRHN	YELDEAVTLQ			
DPB1*02:01	--------E-	-----M----	---GGPM---			
DPB1*04:01	----------	-----M----	---GGPM---			
DPB1*04:02	----------	-----M----	---GGPM---			
DPB1*05:01	----------	-----M----	----------			

### Docking score-based quantitative matrices (DS-QMs) for DP1, DP41, DP42 and DP5

Four libraries, each consisting of 172 peptides (19 amino acids × 9 positions + 1 original ligand), were built. Each peptide was docked separately into the corresponding DP rigid binding site. DS-QMs were derived based on normalized FEB scores, as described in Data and Methods. Dockings were performed at two pH values: 5.0 and 7.0. Over this pH range, only His undergoes protonation/ deprotonation. At pH 5.0, His is protonated and very hydrophilic, yet at pH 7.0 His is neutral and less hydrophilic. The eight DS-QMs (four at pH 5.0 and four at pH 7.0) derived here are given in Additional file 2.

### External validation

A test set comprising 484 peptides known to bind HLA-DP1, HLA-DP41, HLA-DP42 or HLA-DP5, originating from 263 proteins, was used for external validation of the derived DS-QMs. Initially, the sensitivity of the top 5% of the best scored peptides for each position was assessed using DS-QMs calculated at pH 7.0 and pH 5.0. Next, all possible combinations of different positions were evaluated. The most predictive models among all possible combinations between the nine peptide positions are shown in Table 2. It is evident that almost all positions are involved in these highly predictive models, indicating that no peptide positions have a negligible effect on binding. The results also indicate that the models derived at pH 5.0 seem to predict better than those derived at pH 7.0. Moreover, different peptide positions are important for binding at different pH values.

**Table 2:** The most sensitive models for HLA-DP peptide binding prediction at threshold of top 5%

***DP protein***	***pH 7.0***			***pH 5.0***		
	***model***	***sensitivity***	***AUC***	***model***	***sensitivity***	***AUC***
DP1	p2p7p8	0.426	0.865	p3p7p8	0.455	0.860
DP41	p1p2	0.490	0.886	p1	0.497	0.864
DP42	p1p3p4p5p6p7	0.471	0.883	p1p2p3p8p9	0.504	0.900
DP5	p5p6	0.514	0.880	p1p2p5p7p9	0.523	0.883

### Comparison to existing servers for HLA-DP binding prediction

**Figure 2:**
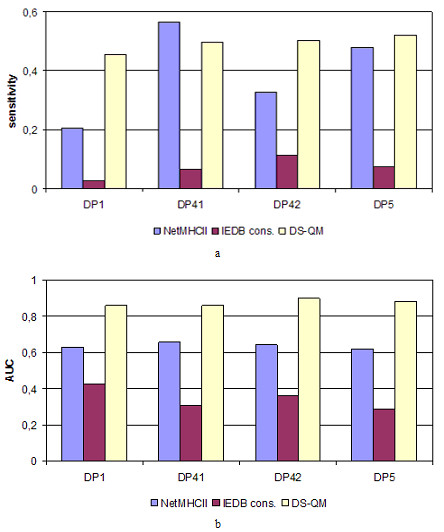
***Sensitivities *** **of the predictions calculated at threshold of top 5% predicted binders (a) and *AUC *** **values (b) by different servers for HLA-DP binding prediction.**

The best performing models derived here were compared to two state-of-the-art servers for MHC class II binding prediction: NetMHCII [27] and IEDB [28]. Both use sequence-based models powered by artificial neural networks (ANN). NetMHCII identifies nonamers, while IEDB works only with 15mers. The tests were performed as follows: protein sequences were converted into sets of overlapping peptides (9mers for NetMHCII and 15mers for IEDB), and the binding score of each peptide was predicted; peptides were ranked according to their binding score, and the top 5% of the ranked peptides were selected and compared to known binders. If the predicted peptide was included in the known binder sequence, it was considered a true predicted binder. The ratio of all true predicted binders to all binders in the corresponding test set defined the *sensitivity* of prediction at the top 5% cut-off. The sensitivities were recorded and compared to our best predicted models at pH 5.0 (Figure 2a). Additionally, servers were compared in terms of *AUC* (Figure 2b). It is evident that our DS-QM models out-performed state-of-the-art servers for DP1, DP42 and DP5 proteins.

### Effect of pH on peptide and protein His residues

**Figure 3:**
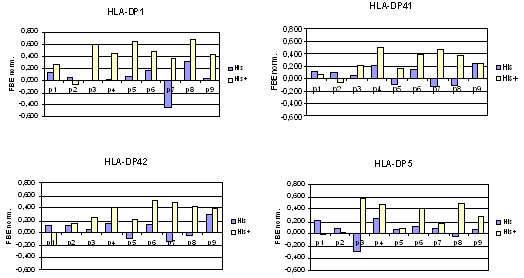
**Normalized FEB values for protonated and nonprotonated His residues at each of the nine peptide binding core positions.** Protonated His is strongly preferred in most positions.

As peptides typically bind to class II MHC proteins in an acidic environment, with a pH between 4.5 and 5.5, dockings were performed at pH 5.0 and pH 7.0, and compared in terms of their predictive ability. Better prediction was found for the DS-QMs derived at pH 5.0. The only amino acid sensitive to pH in the range 5.0 to 7.0 is Histidine. The pK_a_ of the His imidazole is 6.0, thus making His protonated and very hydrophilic at pH 5.0 and unprotonated and less hydrophilic at pH 7.0. The influence of pH on the affinity of peptide binding to HLA-DP proteins has two potential aspects: influence on peptide protonation/deprotonation and influence on protein binding site protonation/deprotonation. Figure 3 summarizes the normalized FEB values for protonated and nonprotonated His residues at each of the nine peptide binding core positions. It is clear that protonated His residues are preferred in most peptide positions (p3 to p9). As the peptide binding site on DP proteins is predominantly negatively charged [29], preference for positively charged His were expected.

**Figure 4:**
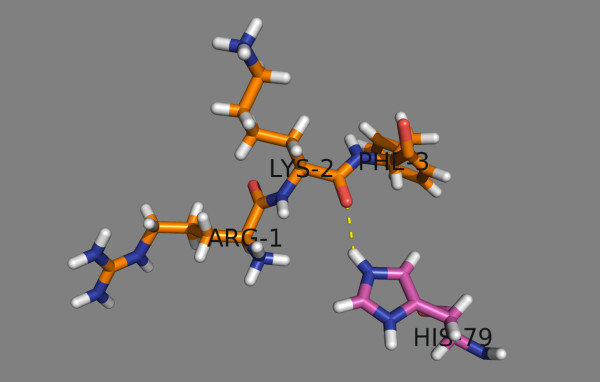
**At pH 5.0 an additional hydrogen bond between the backbone carbonyl oxygen from the peptide position (−1) (here LYS-2) and the imidazole ε-nitrogen of protein His**^**79β **^**is formed.**

Five His residues are present in the HLA-DP binding site: four belong to the α-chain (positions 5, 16, 44 and 79) and one belongs to the β-chain (position 79). All are conserved among the studied DPs. Only His^79β^ contacts the binding peptide in the vicinity of peptide position 2; the other His residues are distant from the binding site. The protonation of His^79β^ allows an additional H-bond to be formed between the backbone carbonyl oxygen belonging to peptide position −1 (the position before p1) and the imidazole ε-nitrogen of His^79β^ (Figure 4). The estimated N-H…O = C bond energy for polypeptides in water environment lies within the range: 1.5 – 2 kcal/mol [30]. This means that the formation of this additional H-bond can increase the binding affinity constant of the peptide-protein complex by over an order of magnitude in the absence of other effects. This may explain the enhanced experimentally-observed equilibrium binding level seen at pH 5.0 [13].

## Discussion

In the present study, molecular docking procedures developed recently for peptide binding prediction to HLA-DP2 protein [15] were significantly extended to include the four most frequent DP proteins [18]: DP1 (DPA1*0201/DPB1*0101), DP41 (DPA1*0103/ DPB1*0401), DP42 (DPA1*0103/DPB1*0402) and DP5 (DPA1*0201/DPB1*0501). The X-ray structure of the peptide – HLA-DP2 protein complex was used as a starting template to model by homology the structure of the four DP proteins. In turn, these were used to generate combinatorial peptide libraries built using the SAAS principle. Peptides were docked into the DP binding site using AutoDock at pH 5.0 and pH 7.0. The resulting scores were recorded, normalized, and used to generate DS-QMs. The predictive ability of these QMs was tested using an external test set and compared to existing servers for DP binding prediction. The models derived at pH 5.0 predict better than those derived at pH 7.0, showing significantly improved predictions for three of the four DP proteins, when compared to current state-of-the-art servers. DS-QMs can recognize 50% of the known binders in the top 5% of predicted peptides. Moreover, a single docking of HLA-DP2 (DPA*0103, DPB1*0201) in complex with a self-peptide derived from the HLA-DR α-chain (pdb code: 3lqz) was analysed using RosettaDock. This characterised more fully the interacting amino acids across the peptide – MHC binding interface, helping identify amino acid preferences at each position of the peptide binding core.

**Figure 5:**
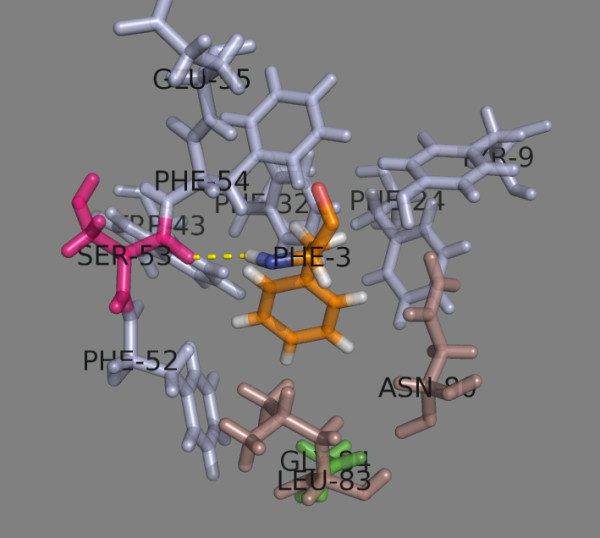
**Peptide binding pocket 1.** The α chain residues are shown in light blue, the β chain residues – in darksalmon. The dimorphic Asp/Gly^84β^ is shown in green. Ser^53α^ (given in magenta) makes a H-bond with peptide position 1 residue Phe (given as PHE-3 in orange).

Peptide binding pocket 1 (p1) consists of 11 residues (Table 3). Ten of them are conserved and only Asp/Gly^84β^ is dimorphic (Figure 5). DP1 and DP5 contain Asp^84β^, while DP41 and DP42 contain Gly^84β^ as does DP2. Aromatic amino acids such as Phe, Tyr, Trp and His, as well as aliphatic Ile and Leu are able to bind into this pocket. Additionally, the Asp^84β^-containing proteins DP1 and DP5 accept positively charged Lys, Arg and His (when is charged at pH 5.0). A hydrogen bond is formed between Ser^53α^ and NH of peptide position 1 (p1) (Table 1).

**Table 3:** Pair energies in peptide binding pocket 1

**DP chain**	**position**	**aa**	**peptide position**	**aa**	**E**_**total**_	**E**_**atr**_	**E**_**rep**_	**E**_**sol**_	**E**_**hbnd**_	**E**_**pair**_
A	9	Tyr	1	Phe	0.02	−0.4	0	0.41	0	0
A	24	Phe	1	Phe	−0.46	−0.45	0	−0.01	0	0
A	32	Phe	1	Phe	−1.69	−1.64	0	−0.05	0	0
A	43	Trp	1	Phe	−0.01	−0.13	0	0.12	0	0
A	52	Phe	1	Phe	−0.39	−0.42	0	0.04	0	0
A	53	Ser	1	Phe	−0.88	−0.50	0	0.33	−0.71	0
A	54	Phe	1	Phe	−0.75	−1.50	0.41	0.34	0	0
A	55	Glu	1	Phe	0.01	−0.01	0	0.02	0	0
B	80	Asn	1	Phe	−0.12	−1.52	0.03	1.38	0	0
B	83	Leu	1	Phe	−0.11	−0.15	0	0.04	0	0
**B**	**84**	**Gly**	1	Phe	−0.17	−0.21	0	0.04	0	0
**sum**					**−4.55**	**−6.93**	**0.44**	**2.66**	**−0.71**	**0**

Polymorphic residues are given in bold. *E*_*tot*_ corresponds to the sum of all energies between the pair residues; *E*_*atr*_ and *E*_*rep*_ are the Lennard – Jones attractive and repulsive energies, respectively; *E*_*sol*_ – the solvatation energy; *E*_*hbnd*_ – energy of hydrogen bonding per residue; *E*_*pair*_ – statistics-based pair term.

**Figure 6:**
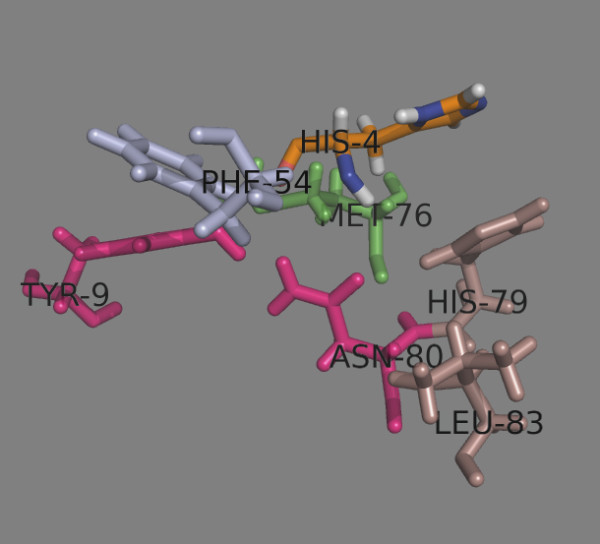
**Peptide position 2.** The α chain residues are shown in light blue, the β chain residues – in darksalmon. The dimorphic Met/Val^76β^ is shown in green. Tyr^9α^ and Asn^80β^ (given in magenta) make H-bonds with p2 residue His (given as HIS-4 in orange).

The peptide position 2 (p2) makes contacts with 6 residues of the binding site (Table 4), 5 of them are conserved, one (Met/Val^76β^) is dimorphic (Figure 6). Only DP1 contains Val^76β^, the remaining DPs have Met^76β^. The p2 side chain protrudes up the binding site close to the β chain and a variety of amino acids are well situated here. His at p2 makes H-bonds with Tyr^9α^ and Asn^80β^, and salt bridges with His^79β^ and Asn^80β^ (Table 4). A π-π stacking of aromatic rings explains the preference of aromatic residues here [15]. Protonated His is not favored here.

**Table 4:** Pair energies in peptide position 2

**DP chain**	**position**	**aa**	**peptide position**	**aa**	**E**_**total**_	**E**_**atr**_	**E**_**rep**_	**E**_**sol**_	**E**_**hbnd**_	**E**_**pair**_
A	9	Tyr	2	His	−0.49	−0.69	0	0.59	−0.39	0
A	54	Phe	2	His	−0.16	−0.25	0	0.09	0	0
**B**	**76**	**Met**	2	His	−0.57	−0.63	0	0.06	0	0
B	79	His	2	His	−1.45	−2.45	0.03	1.13	0	−0.16
B	80	Asn	2	His	−1.94	−1.62	0	1.73	−1.95	−0.10
B	83	Leu	2	His	−0.14	−0.19	0	0.05	0	0
**sum**					**−4.75**	**−5.83**	**0.03**	**3.65**	**−2.34**	**−0.26**

Polymorphic residues are given in bold.

**Figure 7:**
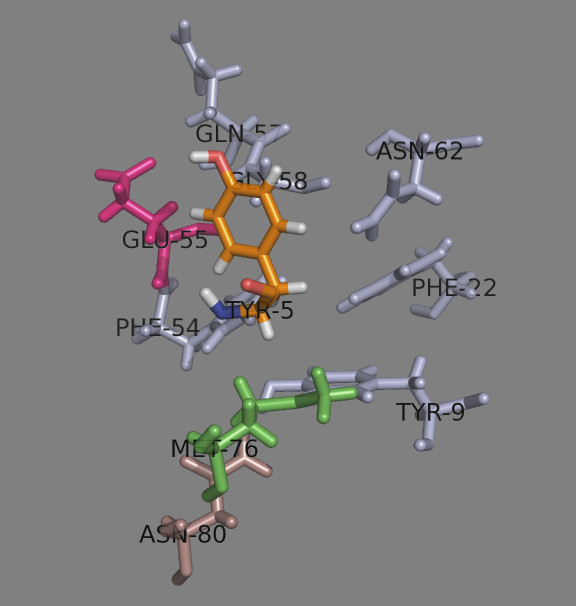
**Peptide position 3.** The α chain residues are shown in light blue, the β chain residues – in darksalmon. The dimorphic Met/Val^76β^ is shown in green. Glu^55α^ (given in magenta) makes a H-bond with p3 residue Tyr (given as TYR-5 in orange).

The side chain of peptide position 3 (p3) protrudes up of the binding site close to α chain. It contacts 7 α-chain residues and 2 β-chain residues, one of which is the dimorphic Met/Val^76β^ (Table 5). Glu^55α^ makes a hydrogen bond with Tyr OH-group (Figure 7). The amino acid preferences here are quite uniform for the four DPs: Tyr, Trp, Phe, Pro and the positively charged Arg. Met^76β^-containing DPs (DP41, DP42 and DP5) accept a protonated His here.

**Table 5:** Pair energies in peptide position 3

**DP chain**	**position**	**aa**	**peptide position**	**aa**	**E**_**total**_	**E**_**atr**_	**E**_**rep**_	**E**_**sol**_	**E**_**hbnd**_	**E**_**pair**_
A	9	Tyr	3	Tyr	−0.53	−0.67	0	0.14	0	0
A	22	Phe	3	Tyr	−0.03	−0.03	0	0	0	0
A	54	Phe	3	Tyr	−0.75	−0.77	0.03	0	0	0
A	55	Glu	3	Tyr	−0.77	−0.93	0	0.79	−0.63	0
A	57	Gln	3	Tyr	0	−0.01	0	0.01	0	0
A	58	Gly	3	Tyr	−0.80	−1.11	0	0.32	0	0
A	62	Asn	3	Tyr	0.01	−0.18	0	0.18	0	0
**B**	**76**	**Met**	3	Tyr	−0.28	−0.40	0	0.11	0	0
B	80	Asn	3	Tyr	0.01	−0.07	0	0.08	0	0
**sum**					**−3.14**	**−4.17**	**0.03**	**1.63**	**−0.63**	**0**

Polymorphic residues are given in bold.

**Figure 8:**
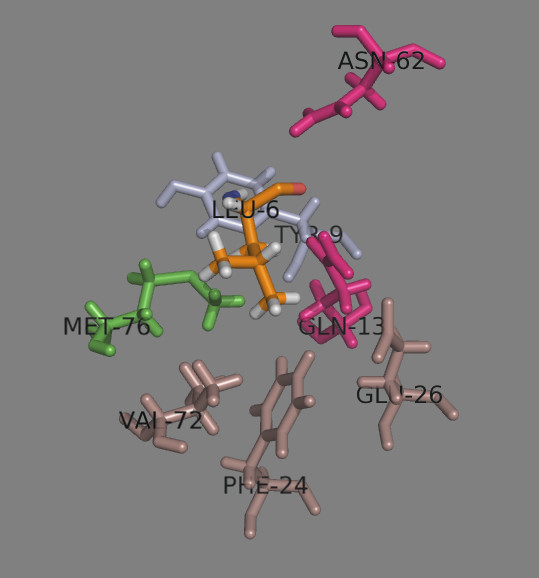
**Peptide binding pocket 4.** The α chain residues are shown in light blue, the β chain residues – in darksalmon. The dimorphic Met/Val^76β^ is shown in green. Asn^62α^ and Gln^13β^ (given in magenta) make H-bonds with p4 residue Leu (given as LEU-6 in orange).

Binding pocket 4 (p4) is large, shallow and negatively charged due to the presence there of Glu^26β^, Glu^68β^ and Glu^69β^[10]. It strongly attracts positively charged amino acids such as Arg, Lys and protonated His. Leu, Tyr, Trp and Phe are also well accepted here. Asn^62α^ and Gln^13β^ make H-bonds with Leu4 (Table 6 and Figure 8). Glu^68β^ and Glu^69β^ are not shown to make contacts to p4, as Leu does not fill the pocket [10]. Surprisingly, Glu, Gln and Asn also fit well into this pocket making H-bonds with Asn^62α^ and Gln^13β^.

**Table 6:** Pair energies in peptide binding pocket 4

**DP chain**	**position**	**aa**	**peptide position**	**aa**	**E**_**total**_	**E**_**atr**_	**E**_**rep**_	**E**_**sol**_	**E**_**hbnd**_	**E**_**pair**_
A	9	Tyr	4	Leu	−0.02	−0.04	0	0.03	0	0
A	62	Asn	4	Leu	−1.05	−0.72	0	0.81	−1.13	0
B	13	Gln	4	Leu	0.18	−1.78	0.71	1.90	−0.66	0
B	24	Phe	4	Leu	−0.37	−0.40	0.11	−0.08	0	0
B	26	Glu	4	Leu	0.08	−0.10	0	0.19	0	0
B	72	Val	4	Leu	−0.76	−0.62	0.11	−0.25	0	0
**B**	**76**	**Met**	4	Leu	−0.86	−0.88	0.16	−0.14	0	0
**sum**					**−2.80**	**−4.54**	**1.09**	**2.46**	**−1.79**	**0**

Polymorphic residues are given in bold.

**Figure 9:**
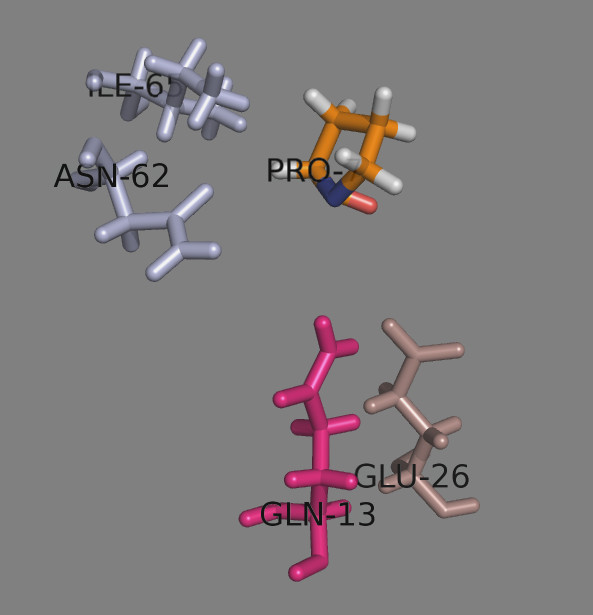
**Peptide position 5.** The α chain residues are shown in light blue, the β chain residues – in darksalmon. No polymorphism exists here. Gln^13β^ (given in magenta) make H-bonds with p5 residue Pro (given as PRO-7 in orange).

Position 5 (p5) protrudes from the binding cleft but it is still in close proximity to the negatively charged residues Glu^26β^, Glu^68β^ and Glu^69β^. This explains the observed preferences for the positively charged Arg, Lys and protonated His and the disinclination for Asp and Glu. Pro at p5 hydrogen bonds to Gln^13β^ and contacts Asn^62α^, Ile^65α^ and Glu^26β^ (Table 7 and Figure 9). Phe and Trp are also well accepted at p5. No polymorphism exists here (Table 2).

**Table 7:** Pair energies in peptide position 5

**DP chain**	**position**	**aa**	**peptide position**	**aa**	**E**_**total**_	**E**_**atr**_	**E**_**rep**_	**E**_**sol**_	**E**_**hbnd**_	**E**_**pair**_
A	62	Asn	5	Pro	0.38	−0.86	0.25	0.99	0	0
A	65	Ile	5	Pro	−0.29	−0.21	0	−0.08	0	0
B	13	Gln	5	Pro	−0.06	−0.39	0	0.36	−0.02	0
B	26	Glu	5	Pro	0.04	−0.04	0	0.08	0	0

No polymorphic residues exist here.

**Figure 10:**
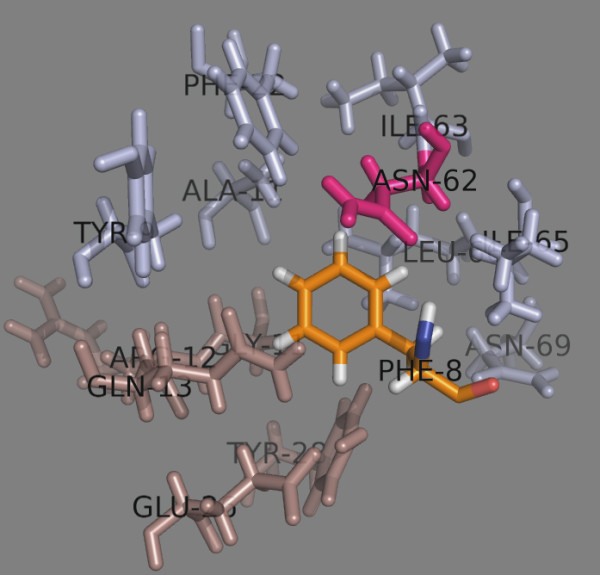
**Peptide binding pocket 6.** The α chain residues are shown in light blue, the β chain residues – in darksalmon. No polymorphism exists here. Asn^62α^ (given in magenta) makes an H-bonds with p6 residue Phe (given as PHE-8 in orange).

Binding pocket 6 (p6) is deep and formed by 8 residues from the α-chain and 5 residues from the β-chain (Table 8 and Figure 10). Asn^62α^ makes an H-bond with the NH of Phe6. No polymorphism exists here (Table 2) and that makes the amino acid preferences at this pocket uniform for the five DPs. Phe, Tyr, Trp and His (protonated and nonprotonated) are well accepted here. Lys and Arg also fit well.

**Table 8:** Pair energies in peptide binding pocket 6

**DP chain**	**position**	**aa**	**peptide position**	**aa**	**E**_**total**_	**E**_**atr**_	**E**_**rep**_	**E**_**sol**_	**E**_**hbnd**_	**E**_**pair**_
A	9	Tyr	6	Phe	−0.01	−0.04	0	0.03	0	0
A	11	Ala	6	Phe	−0.31	−0.36	0	0.05	0	0
A	22	Phe	6	Phe	−0.17	−0.16	0	0	0	0
A	62	Asn	6	Phe	−1.09	−2.44	1.00	1.74	−1.39	0
A	63	Ile	6	Phe	−0.01	−0.01	0	0	0	0
A	65	Ile	6	Phe	−1.15	−1.38	0.02	0.22	0	0
A	66	Leu	6	Phe	−0.76	−1.33	0.77	−0.21	0	0
A	69	Asn	6	Phe	0.02	−0.05	0	0.06	0	0
B	11	Gly	6	Phe	−0.66	−0.82	0	0.17	0	0
B	12	Arg	6	Phe	−0.21	−0.29	0	0.08	0	0
B	13	Gln	6	Phe	−0.31	−1.56	0	1.25	0	0
B	26	Glu	6	Phe	−0.03	−0.06	0	0.04	0	0
B	28	Tyr	6	Phe	−0.50	−1.43	0.35	0.58	0	0
**sum**					**−5.18**	**−9.93**	**2.14**	**4.01**	**−1.39**	**0**

No polymorphic residues exist here.

**Figure 11:**
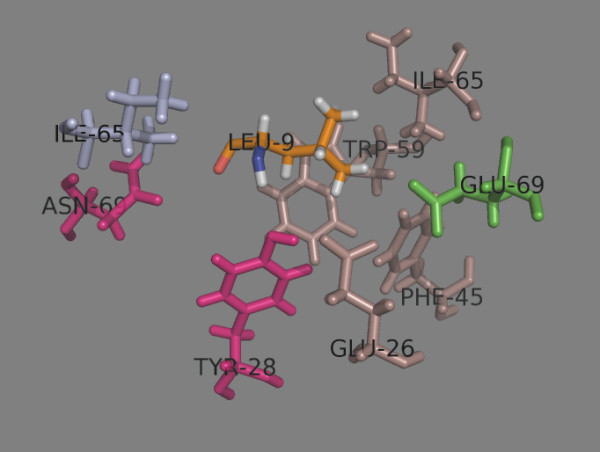
**Peptide position 7.** The α chain residues are shown in light blue, the β chain residues – in darksalmon The dimorphic Glu/Lys^69β^ is shown in green. Asn^69α^ and Tyr^28β^ (given in magenta) make H-bonds with p7 residue Leu (given as LEU-9 in orange).

The side chain of position 7 (p7) lies tangentially to the binding site and is oriented towards the β-chain (Table 9). It is considered to be a secondary anchor position for some MHC class II proteins [31,32]. NH and CO of Leu7 make H-bonds with Tyr^28β^ and Asn^69α^, respectively. The p7 side chain makes contacts with Ile^65α^, Glu^26β^, Phe^45β^, Trp^59β^, Ile^65β^ and Glu/Lys^69β^ (Figure 11). Aliphatic residues are well accepted here. Additionally, Asp is preferred by Lys^69β^-containing DP proteins. Position 69β is dimorphic: DP1, DP41, DP42 and DP5 have Lys^69β^, while DP2 has Glu^69β^. Protonated His is accepted better here than the unprotonated form.

**Table 9:** Pair energies in peptide position 7

**DP chain**	**position**	**aa**	**peptide position**	**aa**	**E**_**total**_	**E**_**atr**_	**E**_**rep**_	**E**_**sol**_	**E**_**hbnd**_	**E**_**pair**_
A	65	Ile	7	Leu	−0.24	−0.34	0	0.10	0	0
A	69	Asn	7	Leu	−1.11	−0.66	0	0.76	−1.21	0
B	26	Glu	7	Leu	0.15	−0.47	0	0.62	0	0
B	28	Tyr	7	Leu	−1.73	−1.36	0	0.87	−1.24	0
B	45	Phe	7	Leu	−0.34	−0.29	0	−0.05	0	0
B	59	Trp	7	Leu	−1.48	−1.87	0.13	0.26	0	0
B	65	Ile	7	Leu	−0.33	−0.24	0	−0.10	0	0
**B**	**69**	**Glu**	7	Leu	0.08	−0.25	0	0.33	0	0
**sum**					**−5.00**	**−5.48**	**0.13**	**2.79**	**−2.45**	**0**

Polymorphic residues are given in bold.

**Figure 12:**
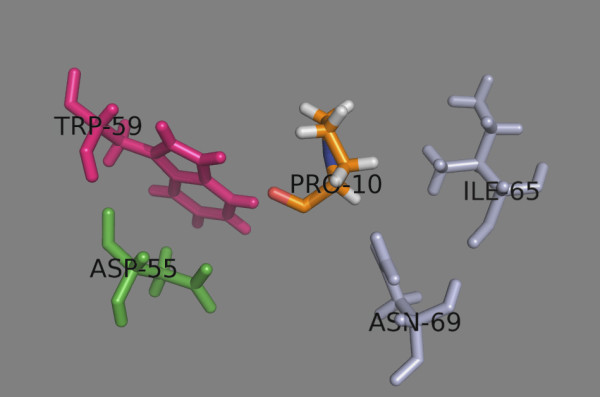
**Peptide position 8.** The α chain residues are shown inlight blue, the β chain residues – in darksalmon. The trimorphic Ala/Asp/Glu^55β^ is shown in green. No H-bonds are made with p8 residue Pro (given as PRO-10 in orange).

Position 8 (p8) is solvent-exposed, yet shows preference for a variety of peptide residues: Trp, Tyr, Pro, Arg, Asn, Gly, Ala, His. Pro8 makes favourable contacts with Ile^65α^ and Trp^59β^ and disfavoured contacts with Asn^69α^ and Asp^55β^ (Table 10 and Figure 12). Position 55β is polymorphic: DP1 and DP41 contain Ala; DP2 and DP42 have Asp; and DP5 has Glu. However, this position is situated far from the side chain of p7 and does not influence the preferences there. Protonated His is preferred here.

**Table 10:** Pair energies in peptide position 8

**DP chain**	**position**	**aa**	**peptide position**	**aa**	**E**_**total**_	**E**_**atr**_	**E**_**rep**_	**E**_**sol**_	**E**_**hbnd**_	**E**_**pair**_
A	65	Ile	8	Pro	−0.14	−0.13	0	−0.01	0	0
A	69	Asn	8	Pro	0.05	−0.73	0	0.78	0	0
**B**	**55**	**Asp**	8	Pro	0.03	−0.02	0	0.05	0	0
B	59	Trp	8	Pro	−0.19	−0.51	0	0.32	0	0
**sum**					**−0.25**	**−1.39**	**0**	**1.14**	**0**	**0**

Polymorphic residues are given in bold.

**Figure 13:**
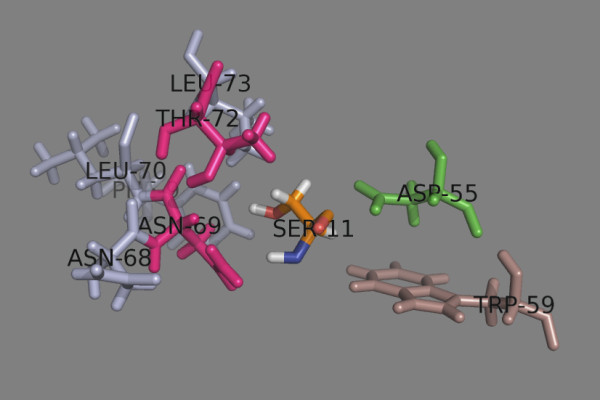
**Peptide binding pocket 9.** The α chain residues are shown in light blue, the β chain residues – in darksalmon. The trimorphic Ala/Asp/Glu^55β^ is shown in green. Asn^69α^ and Thr^72α^ (given in magenta) make H-bonds with p9 residue Ser (given as SER-11 in orange).

Binding pocket 9 (p9) is formed from Asn^68α^, Asn^69α^, Leu^70α^, Thr^72α^, Leu^73α^, Phe/Tyr^9β^, Ala/Asp/Glu^55β^ and Trp^59β^ (Table 11 and Figure 13). It accepts large aromatic, polar, and even charged residues [10]. The side chain of p9 is oriented towards the α-chain. Ser9 is too short to fill the pocket. It makes H-bonds with Asn^69α^ and Thr^72α^. Phe, Tyr, Trp, His fit well into this pocket. The Asp/Glu^55β^-containing DPs accept Arg and protonated His.

**Table 11:** Pair energies in peptide binding pocket 9

**DP chain**	**position**	**aa**	**peptide position**	**aa**	**E**_**total**_	**E**_**atr**_	**E**_**rep**_	**E**_**sol**_	**E**_**hbnd**_	**E**_**pair**_
A	68	Asn	9	Ser	−0.02	−0.05	0	0.07	0	−0.04
A	69	Asn	9	Ser	−0.9	−1.73	0.18	1.99	−1.16	−0.18
A	70	Leu	9	Ser	0	−0.01	0	0.01	0	0
A	72	Thr	9	Ser	0.59	−0.68	0.98	1.13	−0.78	−0.07
A	73	Leu	9	Ser	−0.15	−0.23	0	0.08	0	0
**B**	**9**	**Phe**	9	Ser	0	0	0	0	0	0
**B**	**55**	**Asp**	9	Ser	−0.03	−0.40	0	0.46	0	−0.09
B	59	Trp	9	Ser	−0.03	−0.04	0	0.01	0	0
**sum**					**−0.54**	**−3.14**	**1.16**	**3.75**	**−1.94**	**−0.38**

Polymorphic residues are given in bold.

The influence of pH on the affinity of peptides binding to HLA-DP has two main aspects: influence on peptide protonation/deprotonation and influence on protein binding site protonation/deprotonation. At pH 5.0, His is positively charged and it is preferred at peptide positions 3 to 9. Among the five His residues in the HLA-DP binding site, only the protonation state of His^79β^ affects peptide binding. At pH 5.0 an additional hydrogen bond is formed between the backbone carbonyl oxygen of the peptide position before p1 (p-1) and the imidazole ε-nitrogen of His^79β^ (Figure 4). This H-bond increases the peptide binding affinity by more than 3 orders of magnitude, perhaps explaining the higher experimentally-observed equilibrium binding level seen at pH 5.0 [30]. The peptide-protein association rate constants greatly increases at pH 5.0 (~ 40-fold), while the dissociation rates are almost unchanged in the pH range 5.0 – 7.0 [13]. Thus, one may speculate that the peptide-protein complex formed in the acidic environment of endosomes will also be stable in the neutral environment of the cell surface.

## Conclusion

For peptide binding to the four most frequent HLA-DP proteins (DP1, DP41, DP42 and DP5), statistically the DS-QMs derived through molecular docking simulations at pH 5.0 gave better predictions than those derived at pH 7.0 and performed better than current state-of-the-art servers for MHC binding prediction. Clear differences are observed in our X-ray-based protein-peptide models: an additional hydrogen bond is formed between the backbone carbonyl oxygen belonging to the peptide position before p1 and the protonated ε-nitrogen of His^79β^. This additional hydrogen bond may provide additional stabilization for all peptide regardless of their sequences, provided that they have a sufficiently long N-terminal extension. Protonated His residues make favourable interactions at most of the peptide binding core positions.

## Abbreviations

QM: Quantitative matrix; DS-QM: Docking score-based quantitative matrix; SAAS: Single amino acid substitution; FEB: Free energy of binding.

## Competing interests

The authors declare that they have no competing interests.

## Authors’ contribution

IrDo designed and supervised the study and drafted the manuscript. AP performed the homology modelling and molecular dockings. IvDi performed the external validation. DRF advised on the study and helped with the writing of the manuscript. All authors revised and approved its final version.

## Supplementary Material

Additional file 1 **Test set of known HLA-DP1, DP41, DP42 and DP5 binders.** The file contains a test set of 484 known peptide binders to DP proteins, parent protein NCBI GI numbers and IC50 values. It was derived from Immune Epitope Database (June 2011 release). (XLS 57 kb) Click here for file

Additional file 2 **DS-QMs for peptide binding prediction to HLA-DP1, DP41, DP42 and DP5 proteins.** The file contains 8 DS-QMs: two for each HLA-DP protein derived at pH 5.0 and pH 7.0, respectively. Non-binding amino acids were assigned a binding score of −10. Click here for file
